# Undergraduate students with and without mental health concerns have different perceptions of disclosing mental health challenges to instructors

**DOI:** 10.1371/journal.pone.0315155

**Published:** 2025-03-25

**Authors:** J. Michael Sizemore III, Bailey Von der Mehden, Elisabeth E. Schussler

**Affiliations:** 1 Department of Psychology, University of Tennessee, Knoxville, Tennessee, United States of America; 2 Department of Ecology and Evolutionary Biology, University of Tennessee, Knoxville, Tennessee, United States of America; Texas Tech University Health Sciences Center, UNITED STATES OF AMERICA

## Abstract

Many undergraduates report having mental health concerns (MHC), which can reduce academic success. Students with MHC are encouraged to seek help from their instructors but may not because of perceived negative reactions by instructors and peers. This suggests stigma about MHC may differentially impact help-seeking between students with and without MHC, yet disclosure perceptions between these groups have not been investigated. This study surveyed students with and without MHC in the same classes about their hypothetical disclosure of MHC. Students in several introductory biology classes were asked whether they identified as having MHC, whether they would or would not hypothetically disclose MHC to an instructor, and why. Thematic analysis identified reasons underlying student disclosure choices, which were sorted into the three beliefs of the Theory of Planned Behavior: attitudes, subjective norms, and behavioral control. Of the 803 respondents, 50% self-identified as having MHC. Students with MHC were less likely to hypothetically disclose their MHC to an instructor than students without MHC. Students with and without MHC who said they would disclose gave similar reasons aligned with attitude beliefs. Students with MHC who said they would not disclose perceived that the instructor wouldn’t care (attitude beliefs). Students without MHC who would not disclose were concerned about keeping their MHC private (subjective norms beliefs). Students without MHC who said ‘it depends’ indicated more concerns about impact on their course performance (attitude) than students with MHC. This research found different perceptions of disclosure between students with and without MHC in these courses and suggested that students with MHC focus more on negative instructor reactions, while those without MHC focus on privacy and performance. These different perceptions may contribute to students with MHC perceiving disclosure as a negative social cost versus a positive academic benefit.

## Introduction

A growing number of undergraduate students across the United States are experiencing mental health concerns [1]. Mental health concerns (MHC) encompass many different conditions with varying degrees of severity such as anxiety disorders, depression, post-traumatic stress disorder, and neurodevelopmental disorders, to name a few [2]. Anxiety and depression are some of the most common undergraduate MHC. A national survey of multiple institutions found that 34.9% of undergraduates reported being diagnosed with anxiety and 27% reported being diagnosed with depression, including major depression, persistent depressive disorder, and disruptive mood disorder [3]. Researchers have identified negative relationships between MHC and academic motivation and sense of belonging [4], both of which impact student success.

Undergraduates with MHC are more likely to have negative outcomes in their courses and degree programs. Prior research at our institution found that biology students with higher levels of anxiety also reported a higher likelihood of leaving the biology major [5–6]. Research at other institutions found that students with depression and/or other MHC have less successful academic outcomes [7–8]. Given these negative impacts, students are often encouraged to disclose their MHC to their instructors to seek academic accommodations, such as making up in-class work, extending assignment deadlines, or receiving make-up exams. However, these academic benefits can be perceived by some students as having costs that impact their decisions to disclose.

MHC are one example of a concealed stigmatized identity (CSI), which has been associated with perceived disclosure risks [9]. CSIs are student identities that can be hidden, but if revealed may change the way people view them and their abilities [9]. One study, for example, found that students with MHC avoided requesting accommodations because they feared stigmatization by their teachers or other students [10]. A study by Busch and colleagues [11] documented that students with depression were concerned about being treated negatively or viewed as making an excuse by their instructor. Thus, students with MHC must weigh the academic benefits of disclosure with the perceived personal costs when considering whether to disclose their needs and request an accommodation.

These disclosure costs and benefits are weighed by students in a classroom context that is increasingly evenly split between students with and without MHC [1,3]. And even though awareness about MHC is higher than in the past, almost half of students with MHC perceive stigma from others [1]. Yet, studies on disclosure of MHC typically only focus on perceptions of disclosure by students with MHC and not the perceptions of students without MHC in the same contexts, making direct comparisons of perceptions difficult. To directly compare their perceptions, we asked students with and without MHC in several introductory biology classes about a hypothetical disclosure scenario to investigate whether beliefs about disclosure, including attitudes, subjective norms, and behavioral control, differed among these students. If these beliefs do differ, it confirms perceptions of differences by students with MHC and informs the types of barriers that students with MHC face as they weigh disclosure decisions.

### Factors impacting MHC disclosure

Institutions of higher education recognize that MHC can create barriers to student success. To lower these barriers, they encourage students to seek accommodations to reduce the impact of MHC on their academic outcomes. The process of getting an accommodation generally involves two steps: (1) working with the institutional disability office to document MHC needs, and (2) asking a professor for an accommodation [12]. As the number of students with MHC have risen in the college population, so has the number of students seeking institutional assistance [1,13]. Yet, there is still a large proportion of students who do not request or receive support for their MHC [13]. For some students, the difficulty of navigating the healthcare system to receive documentation of their MHC precludes an official accommodation process [10]. Even with documentation, however, there are still many barriers to disclosure.

Many students face logistical challenges related to institutional accommodation or treatment. Students in one study were unable to articulate what their needs were and therefore were unable to advocate for themselves at their institutional disability office; others did not think the accommodation they would receive would be helpful [14]. Interviews of 33 engineering students identified challenges such as ease in accessing treatment, cost, timeliness, and getting the resources needed to treat their MHC [15]. Even after institutional approvals, the student still must ask their professor to honor the accommodation [12]. These logistical challenges are not the only barriers to disclosure, however; many students also struggle with the way others view their MHC.

As stated previously, mental illness is one example of a CSI which include aspects of an individual’s identity that are not visibly identifiable and have negative stereotypes or attributes ascribed to them [16–18]. Despite increased awareness about MHC and some decline in perceived stigma, one study found 46% of surveyed students with MHC still perceived stigma about mental illness [1]. These perceptions may cause students to view disclosure as having a social cost because of fears of discrimination or disclosure reactions [16]. Another study found that students chose not to disclose their MHC to instructors or peers to distance themselves from negative stereotypes about MHC (such as not being capable) and maintain a preferred social identity [19]. This indicates that despite the encouragement to disclose their need for accommodations and receive academic assistance, many students are weighing the benefits they might receive against the social costs disclosure might incur.

Two studies illuminated how college students with MHC weigh the potential supports and negative impacts of disclosure. Busch and colleagues studied how undergraduate students with depression viewed disclosure to their online biology instructors [11]. These students recognized that disclosure of MHC could increase student-instructor communication and add flexibility to their workload and deadlines. However, the perceived cost of disclosing MHC, such as being treated negatively or being viewed as making an excuse, outweighed the benefits for many students. Kranke and colleagues found that students often wanted to maintain autonomy and normality, which contributed to their reluctance to disclose MHC to a professor [20]. However, students were willing to talk about their MHC with a professor when they perceived the instructor was supportive and when they felt their academic performance could be jeopardized [20]. The work of these two studies provided empirical evidence that there are many factors that impact intentions to disclose MHC, and many of those perceived costs and benefits seem to be aligned with theoretical aspects of the Theory of Planned Behavior.

### Theoretical model

The Theory of Planned Behavior (TPB, Table 1) theorizes that the intent to enact a particular behavior is guided by individual beliefs about that behavior [21–23]. These beliefs include an individual’s attitudes, subjective norms, and behavioral control related to the behavior [22]. TPB is an ideal theoretical framework for this study as it is often used by researchers to understand how people make decisions about health-related behaviors [23]. In the context of our study, we are studying students’ intentions to disclose MHC to an instructor, which should be informed by students’ attitudes about disclosing MHC, the subjective norms they perceive about MHC, and their behavioral control beliefs about disclosing MHC (Table 1). Importantly, this theory can be applied to students with and without MHC in our study because we are presenting them with a hypothetical scenario and asking them about their intent to disclose in that situation.

**Table 1:** The Three Beliefs of Ajzen’s Theory of Planned Behavior. The three beliefs guiding the intention to act are shown with examples specific to disclosing MHC to an instructor. These beliefs inform the intention to disclose to an instructor, which is aligned theoretically with the behavior of disclosing.

TPB belief	Beliefs, as contextualized in this study
Attitudes	How does the student feel about disclosing MHC to an instructor?
Subjective Norms	How do others perceive the disclosure of MHC?
Behavioral Control	How comfortable is the student with disclosure of MHC?

‘Attitude’ beliefs are student perceptions about how *they* might feel (positively or negatively) about disclosing their MHC or their perception about the positive or negative outcomes that might arise from the disclosure. A student may believe, for example, that disclosing MHC may result in a flexible deadline for an assignment (a positive outcome), while another student may perceive they would feel shame in disclosing their MHC (negative feeling). ‘Subjective norms’ are beliefs about how an individual thinks *others* will view their MHC disclosure or how others they respect might act in a similar situation. Thus, a student would have a higher intention to disclose MHC if those they respected in their social network (i.e., friends, family, peers) viewed disclosure as a positive and/or acceptable behavior. However, they might be less inclined to disclose if those in their social network do not disclose MHC despite the need to do so. Finally, ‘behavioral control’ is the extent to which an individual thinks they have the agency to disclose MHC, based on their confidence and perceived barriers. A student with high self-confidence about their ability to disclose MHC to an instructor would be more likely to do so. A student who perceives significant situational barriers to disclosure would be less inclined to do so.

A recent study used TPB to investigate disclosures of MHC to instructors among 311 students in a communications course at a small private California University [24]. They found that all three TPB beliefs were related to intention to disclose, but subjective norms beliefs were most strongly related to that outcome. They did not disambiguate the results by those with and without MHC, however. Their findings support that attitude, subjective norms, and behavioral control beliefs are perceptions a student with or without MHC would use to react to the scenario about intention to disclose MHC in this study. Even though beliefs vary by individual, there may be patterns in how particular populations (e.g., students with and without MHC in introductory biology classes) relate beliefs to their intentions (e.g., disclosing MHC). Therefore, we investigated which of the TPB beliefs might drive intent to disclose MHC in our introductory biology population, and whether those beliefs differed among students with and without MHC, to explore potential differences in student perceptions about MHC and its disclosure that might indicate the potential for stigma.

### Rationale

Prior research provided a foundation for investigating the costs and benefits students with MHC perceive when they are weighing disclosure of MHC to an instructor [11,20]. However, one study was specific only to depression and disclosure in an online setting [11], and both studies only probed the perceptions of students with MHC. Our study asked students with and without MHC about their hypothetical intentions to disclose MHC to an instructor and used the reasons for their disclosure choices to determine their TPB beliefs. We posited that students with and without MHC would make different choices about intention to disclose that were driven by different TPB beliefs, with students with MHC mentioning subjective norms more often due to the stigmatized nature of MHC. To explore this hypothesis, we collected data to answer the following research questions:

1) Do students with and without MHC make different choices about hypothetical disclosure of MHC to an instructor?
2) What are the differences in disclosure reasoning for students with and without MHC?

If disclosure intentions and TPB subjective norms differ between students with and without MHC in introductory biology classes, it would potentially support perceptions of stigma that impact whether students with MHC disclose to their instructors. Our findings suggest the existence of a stigmatized environment related to MHC disclosure and highlight a need for instructors to create a classroom climate that decreases these perceptions to encourage support for all students.

## Methods

### Courses and participants

This study surveyed students in two large introductory biology courses at one research-intensive university in the southeastern United States. One of the courses was introductory biology for non-science majors focused on molecules, cells, and body systems, and the other was introductory biology for majors focused on ecology, evolution, and biodiversity. The non-science majors’ course is taken as a general education credit by students in many majors, but is also an option for students in nursing, kinesiology, and psychology. The majors’ course is the first in a two-course sequence and is typically composed of about 70% STEM majors and 30-40% biology majors.

Surveys were sent to students in five lecture sections of the non-science majors’ course and two lecture sections of the science majors’ course. All the instructors were non-tenure track faculty with several years’ experience teaching introductory biology courses. There were three non-science major lecture sections with 225 students and two with 150 students for a total of 975 students in that course. One of the science major sections had 200 students and one had 225 students for a total of 425. This study was approved by the University of Tennessee, Knoxville, Institutional Review Board under expedited protocol UTK IRB-23-07777-XP. There was a waiver of written consent for the survey; students 18 years old or older indicated consent through a yes/no radio button after reading the consent information.

### Data collection

The data for this study were collected during the fall 2023 semester. The researchers provided instructors of the classes a recruitment email with survey link and consent information and asked them to forward the information to their students. Students clicked the link to the survey, read the consent information, and then clicked yes or no to provide permission to use their data for the research study. The survey was distributed to students on November 20, 2023, and was available until December 5, 2023. Instructors were allowed to incentivize participation by offering one point of class credit; classes had 800-1,000 points total. We utilized Qualtrics Survey software (https://www.qualtrics.com) as the surveying platform.

### Survey instrument

The survey questions (S1 Table) were developed by the authors and reviewed by education research experts for clarity and alignment with the research questions. Students were first asked, “Do you currently identify as having a mental health concern (whether documented or not), e.g., anxiety, generalized anxiety disorder, depression, bipolar disorder, PTSD, panic disorder, suicidal ideation?” to which they answered yes, no, or prefer not to answer. Students were then asked, “Hypothetically or not, if you were experiencing a mental health concern that was or could be impacting a course task (e.g., meeting a course deadline and/or studying for an exam and/or taking an exam), would you disclose the mental health concern to your instructor? Explain Why.” They could answer yes, no, or it would depend. In addition to those questions, there were questions about course type (non-science major or science major course), race and/or ethnicity, college generation status, year in college, gender, and major (answer choices for all demographic questions provided in the S1 Table) that were used solely to describe the sample.

### Data analyses

Partial responses were removed from the dataset, as well as data from students who were not 18, who did not give consent for their responses to be used in the study, and who indicated that they preferred not to respond to the disclosure question. After removing these data, the final sample size was 803 students.

### Do students with and without MHC make different choices about hypothetical disclosure of MHC to an instructor?

We sorted students into those who did or did not identify as having MHC and summed the disclosure choices (yes, no, or prefer not to answer) for each group. Percentages were calculated for each, and a chi-square test was conducted to identify whether there were significant differences in the distribution of disclosure choices between students with and without MHC [25]. Significance was indicated by a p-value less than alpha which was equal to 0.05.

### What are the differences in disclosure reasoning for students with and without MHC?

Students followed up their disclosure choice with a written explanation describing the reason for their choice. Two coders (M.S. and E.S.) analyzed student responses to inductively create categories that could be named and described (codes), in a process called inductive thematic analysis [26]. Inductive creation of codes (versus deductively using pre-existing categories) was selected for this study because there were few studies in a college science context to draw from, and we did not want to constrain student voice into pre-existing categories that may not capture all their perceptions. Therefore, we let participant voice guide the identification of codes and then organized the codes using the framework of TPB beliefs for analysis. There were codes that did not neatly fit in the TPB framework, suggesting this inductive approach was appropriate.

Thematic analysis is an active process of researcher(s) building meaning from a set of data, and as such the identities of the researchers can impact the analysis [26]. To honor this, we reflect here on the roles and positionalities of each researcher in this study. M.S. is a neuroscience major interested in mental health and well-being who conducted research in the lab of E.S. starting in August 2023. He had not conducted thematic analysis before. E.S. has conducted biology education research on introductory biology classes for over 15 years, including work on the impacts of anxiety, and numerous studies employing inductive thematic analysis. M.S. spent Fall 2023 studying the literature about MHC and education research methods such as thematic analysis and developing the methods for the study. He, E.S., and B.V.D.M. met weekly to discuss all aspects of design and data collection. In preparation for coding in Spring 2024, M.S. and E.S. discussed the principles necessary for reliable coding, including equal voice in the process, and objectively assessing strength of arguments for coding decisions to reduce authoritative bias. B.V.D.M. is a graduate student in the lab of E.S. who also conducts research on introductory biology classes; she provided objective feedback about the clarity of the codes that were generated and aided in the process of sorting the codes into TPB belief categories. The research team closely monitored and discussed their interpretation of the data during the analysis phase.

To conduct inductive thematic analysis, the two coders started by independently reading student responses and taking notes on common ideas mentioned by the students [26]. The researchers then discussed their notes to create an initial codebook which is a document guiding how to sort student responses into categories (called codes). Codebooks are iteratively refined through rounds of testing on subsets of the dataset until the coders reach a minimum percent agreement on their independent assignment of codes. After each round, the coders work together to expand or collapse codes and edit descriptions for codes that were challenging to agree upon. This continues until a minimum intercoder agreement percentage is reached; for us, this minimum was set at 70% [27]. Once this level of agreement is reached, the codebook is considered reliable enough for coders to use independently. In this study, the researchers went through three rounds of independently coding 50 student responses, identifying agreements and disagreements, discussing the code assignments, and revising the codebook before the codebook was finalized.

M.S. and E.S. independently coded 400 student responses and checked intercoder agreement on this portion of the data, and then E.S. independently coded the last 403 responses. For analysis, each student response was considered the coding frame and was assigned as many codes as needed to represent the ideas being conveyed by the student. For the portion of the dataset the coders both coded, agreement among code assignment (as assessed by individual codes and not the entire unit) was 72%. The coders met to discuss the codes they did not agree on and revised coding decisions to reach 100% agreement. Having met the standard of 70% agreement, E.S. coded the last 403 responses independently due to more availability at that time. Throughout the implementation and reporting of the thematic analysis, the authors adhered to standards of validity (sometimes referred to as rigor) such as systematic and consistent coding, coherent and logical results that actively interpret the data, and rich description of the analytic process [26].

To align the codes identified through inductive analysis with the three beliefs of TPB (attitude, subjective norms, behavioral control), all authors read the theoretical descriptions of each belief and independently placed each code from the codebook into a belief group. After viewing and discussing this initial sorting, the research group decided to create belief prompts to aid in the placement of the codes. For attitude beliefs, the prompts were: (1) Disclosure could make me feel ___ and (2) If I disclose, this could happen ___. For subjective norms beliefs the prompts were: (1) Other people think that disclosing is ___ and (2) People I know ___. For behavioral control beliefs, the prompts were: (1) I feel ___ about my capability to disclose or (2) Disclosure will be easier or harder because of ___. The authors used these prompts to sort the codes again and discussed their placement to consensus. The team agreed that there were two inductive codes that could be aligned with two TPB beliefs; these were called “hybrid” codes.

To answer the second research question, the data were sorted into students with and without MHC, and then each of these groups was subdivided based on their disclosure intention: yes, no, or it would depend. Within each of these six groups, the percentage of students who mentioned each of the codes was calculated by summing the code incidence and dividing by the number of students in the group. In presenting these data, we highlighted > 10% differences in code prevalence between students with and without MHC and removed any codes with less than 5% prevalence across all six groups.

## Results

There were 803 respondents to the survey (Table 2). Of these, 422 were from the non-science majors’ course (43% response rate) and 381 were from the science majors’ course (90% response rate). The sample overall was majority women (76.6%), white (81.7%), continuing generation (85.6%), first-year students (66.3%). Across the entire sample, 50% of the respondents self-reported MHC; 49% of the students in the non-science majors’ course self-identified as having MHC and 50% in the science majors’ course.

**Table 2:** Demographic characteristics of the survey respondents.

Sociodemographic Characteristics		% (N = 803)
Gender	Woman	76.6
	Man	22.6
	Genderqueer	0.2
	Preferred not to respond	0.5
Race and/or Ethnicity	Asian	4.6
	Black	3.4
	Hispanic, Latine, or Spanish origin	2.9
	Multiracial	5.9
	White	81.7
	Other	0.5
	Preferred not to respond	0.9
College Generation	Continuing generation	85.6
	First-generation	13.3
	Does not know or preferred not to respond	1.0
Year in College	First	66.3
	Second	23.4
	Third	6.7
	Fourth	2.2
	Fifth +	0.9
	Preferred not to respond	0.5
Major	STEM major	58.0
	Non-STEM Major	41.4
	Preferred not to respond	0.5

### Do students with and without MHC make different choices about hypothetical disclosure of MHC to an instructor?

For the hypothetical question of whether students would disclose MHC to an instructor, student responses (yes, no, or it would depend) differed significantly between students with and without self-reported MHC (*X*²(2, *N* =  803) =  53.47; p <  0.001). Students with and without MHC were almost identical in their selection of the response ‘it would depend’ (56% and 57%, respectively) (Table 3). However, those who self-identified as having MHC were less likely to say they would disclose MHC (‘yes’) to an instructor (9%) compared with those without MHC (25%). For those who self-identified as having MHC, 35% said they would not disclose to their instructor, while 18% of those without MHC said they would not disclose to an instructor.

**Table 3:** Comparison of disclosure choices between students with and without self-reported MHC. Survey responses shown in S2 Appendix.

	Yes MHC (50%, *n* = 398)	No MHC (50%, *n* = 405)
It would depend	56% (*n* = 223)	57% (*n* = 231)
Yes	9% (*n* = 34)	25% (*n* = 100)
No	35% (*n* = 141)	18% (*n* = 74)

### What are the differences in disclosure reasoning for students with and without MHC?

#### Disclosure codes.

The inductive codes from student responses were sorted into the three TPB belief categories and one hybrid category: 1) Attitude beliefs, 2) Subjective Norms, 3) Behavioral Control beliefs, and 4) Hybrid beliefs (Table 4). There were five inductive codes in the attitude beliefs category, two in the subjective norms category, one in the behavioral control beliefs category, and two in the hybrid beliefs category. Brief descriptions of each TPB belief and code, along with an exemplar student quote, are shown in Table 4.

**Table 4:** Inductive codes generated from student responses, organized into TPB beliefs. The codes (numbered) represent common categories about why students would or would not disclose MHC to an instructor. Additional exemplar quotes for each code are shown in the S3 Appendix.

TPB belief category and inductive codes	Exemplar student quote for each code
**ATTITUDE**: how a student felt about disclosing or about the disclosure outcome. Prompts: (1) Disclosure could make me feel ___ and (2) If I disclose, this could happen	
1. Embarrassing - student said disclosing would make them feel ashamed or nervous; said it might make MHC worse because of increased anxiety.	*“I know that many instructors are very understanding, but letting one know you need help or are behind because of mental health almost feels embarrassing.”*
2. Instructor won’t help - student said instructor likely wouldn’t provide help or accommodation, so there was no use in disclosing; some said it may be because they lacked documentation or diagnosis to validate MHC.	*“I feel like a lot of professors would not really do anything about it.”*
3*.* Instructor aware and might help - Student said they would disclose to instructor early in the semester in case they needed help later; thought instructor would help, support, accommodate; they would disclose because mental health is important.	*“I would inform each of my professors about it so we could hopefully do what it takes to get around it.”*
4. Impact on performance - student said they would disclose if it was impacting success, learning, or attendance in a class.	*“It would depend on how much it was impacting the class.”*
5. Instructor may or may not care - student said it would depend on whether they thought the instructor would believe them/ think the concern was valid; or whether they thought the instructor would care, understand, show concern and respect, listen, and take them seriously.	*“But sometimes instructors don’t take that seriously and say that you should’ve known you would struggle and should’ve come to them sooner...”*
**SUBJECTIVE NORMS**: how a student thought others might view their disclosure or act themselves. Prompts: (1) Other people think that disclosing is ___ and (2) People I know ___.	
1. Private to student - student would prefer not to disclose because it is a personal concern, not the instructor’s; student said they would not be comfortable talking about it.	*“I typically think of this as private information that is not to be discussed.”*
2. Perceptions others have - student wouldn’t want to disclose because others may view an accommodation as an excuse or special treatment and then see you differently or “less than” in some way.	*“I would tell my teacher, but would try to meet in person so that they didn’t think it was an excuse and that I was slacking off on that assignment.”*
**BEHAVIORAL CONTROL**: how a student felt about their abilities or barriers to disclosure. Prompts: (1) I feel ___ about my capability to disclose or (2) Disclosure will be easier or harder because of ___.	
1. Approachability of instructor - student said it would depend on how comfortable they were with instructor, how open the instructor is to talking with students.	*“Some professors are more open to helping students...”*
**HYBRID**: combination of two of the TPB belief categories	
1. Severity of MHC - student said they would only disclose if it was having a major impact on attendance; if it was an illness with broad impacts; they often talked about this as a last resort. (hybrid of attitude/ control)	*“It would depend on what I am going through, as some situations can be more severe than others.”*
2. Won’t let it impact - student said there would be no need to disclose because they wouldn’t let it impact their performance or they would deal with the consequences on their own. (hybrid of subjective norms or control)	*“I have had issues before and personally I feel that getting used to them, doing what you can to help, or simply dealing with the consequences is something that has to be done on your own.”*

ATTITUDE codes were beliefs students had about how they might feel if they disclosed their MHC to an instructor or what outcome they might expect. The code ‘embarrassing’ was the perception that having to disclose MHC might make the student feel ashamed or nervous. A few students mentioned that disclosing could make their MHC worse because of the anxiety it would cause them. The code of ‘instructor won’t help’ was when students said they would not disclose to an instructor because they did not think the instructor would do anything; students mentioned that this might be because of strict course policies or their lack of MHC documentation. The code of ‘instructor aware and might help’ was when students said they should disclose their MHC to their instructor because MHC advocacy was important, and the instructor needed to know about their concerns. Many students said specifically that this was important because the instructor would then work to provide resources or assistance to help. The ‘impact on performance’ code was a risk/ benefit assessment the student was making to decide how much class work they could miss before they would need to ask an instructor for help. The code of ‘instructor won’t care’ was based on student perceptions of negative reactions by the instructor in response to their disclosure. Some students talked about the instructor not believing that the MHC was real or that it would cause a problem; some said that the instructor would not show care or respect or take their concern seriously once they disclosed the MHC.

SUBJECTIVE NORMS codes were beliefs students had about how others might view their disclosure or what others they respected might do in the same situation. The code of ‘private to student’ was when students talked about MHC being a personal concern that they would not want to disclose to an instructor. Students often talked about the comfort level of disclosure related to privacy. The code of ‘perceptions others have’ was when students talked about whether disclosure and subsequent accommodation might result in them being viewed by their peers or instructor differently because of the special treatment they had received; they were particularly concerned about it making them appear “less than” others.

BEHAVIORAL CONTROL were beliefs about conditions that made it easier or harder for the student to disclose, or their capability to disclose. The only code in this category was ‘approachability of instructor’ which was when students talked about how open the instructor seemed to be with regards to talking with students about concerns like MHC.

There were two HYBRID codes which were student beliefs that seemed to cut across two of the belief categories above. The code of ‘severity of MHC’ was when the participant talked about their decision to disclose depending on the type of MHC or how severe the MHC was because that would determine how much school they might miss. This could have been based on a perceived outcome, but students also talked about this being out of their control, so they lacked agency. The code of ‘won’t let it impact’ was when students said they would not need to disclose MHC because it was not something to be used as an excuse to not do their work; they would do the work regardless of the MHC. On one hand this was showing agency, but there was also a hint of not wanting to disclose which could reflect perception of a norm.

#### Patterns in disclosure explanations.

**Fig 1:**
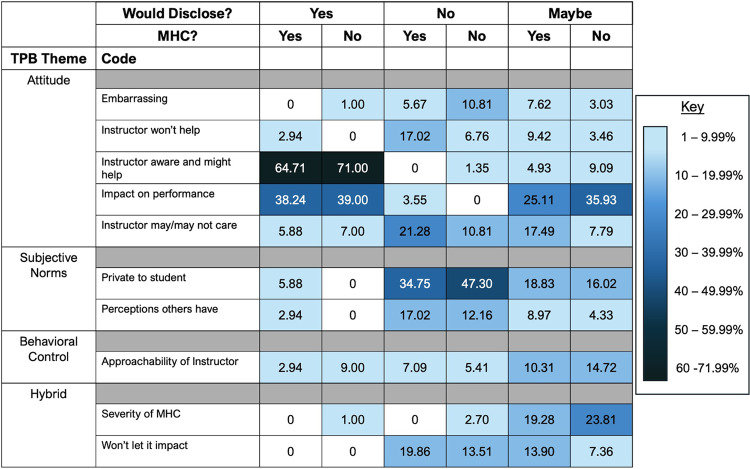
Heat map of disclosure reasoning prevalence by student groups. The heat map shows the relative prevalence of codes explaining student reasoning for their disclosure choices, sorted by disclosure choice and whether they do or do not have MHC. The codes are aligned by the TPB belief categories on the left. Codes are increasingly darker with higher prelevance, as shown by the key on the right. Code frequencies are shown in the S2 Appendix.

The prevalence of each code, aligned with their TPB belief categories and sorted by disclosure choice and MHC status, is shown on a heat map (Fig 1). This allows for two interpretations: (1) which codes and TPB beliefs are driving disclosure choices overall and (2) how students with and without MHC may differ in their disclosure reasoning.

*Disclosure explanations overall -* For each disclosure choice (yes, no, it would depend) there was one most prevalent code. Students who said they would disclose to an instructor mostly talked about the need to proactively make the instructor aware of their needs, which was a TPB attitude belief. For example, one student said, “As someone with anxiety, I always disclose my health concerns with my professors, as I get extra time for tests and quizzes. I let them know at the beginning of the semester....” Students who said they would *not* disclose to an instructor mostly talked about the subjective norms belief of privacy. One student said, “I feel like society does not view it as a valid reason to miss an assignment.” Students who selected ‘it would depend’ as their disclosure choice mostly talked about performance impacts, an attitude belief. One student, for example, said, “I would always disclose this with my professor if I felt it was impacting my performance. My academic success is my priority and it is important to be open if something could be interfering with that.” Although these codes were the most commonly voiced for each disclosure choice, there were differences between students with and without MHC.

*Disclosure reasoning between students with and without MHC -* For students who said they **would disclose** to an instructor, students with and without MHC had similar code percentages suggesting similar beliefs about their disclosure choice. For students who said they **would not disclose**, we highlight three differences. Students without MHC were more likely to say that MHC were private to them (47.30%) in comparison to students with MHC (34.75%). Conversely, students with MHC were more likely to say that the instructor would not care or believe them (21.28%) compared to only 10.81% of students without MHC. One student with MHC voiced, “Many professors do not understand mental illnesses or simply are not concerned about it.” Finally, students with MHC were also more likely to say that the instructor would not help them (17.02%) compared to students without MHC (6.76%). As one student with MHC indicated, “I have always seen these requests ignored despite having a professional diagnosis.” For students who said **disclosure “would depend,”** there was only one code difference: students without MHC were more likely (35.93%) than students with MHC (25.11%) to mention that an impact on their performance was important to their disclosure.

## Discussion

Collegiate courses are increasingly an equal mix of students with and without MHC [1,4]. We posited that students with and without MHC in introductory biology courses would view disclosure intentions differently and have different beliefs about subjective norms that may indicate stigma associated with disclosure for students with MHC. Our prediction that students with and without MHC would have different disclosure intentions was affirmed; students with MHC were less likely to say they would disclose an MHC to an instructor. We found that students who said they would disclose had similar reasons regardless of MHC status. The reasons students would not disclose or were uncertain about it differed across MHC status in ways we did not predict. Students with MHC expressed more concerns about how an instructor would negatively react to their disclosure (attitude belief), while those without MHC were more concerned about privacy (subjective norms belief) or performance impact (attitude belief). These findings provided evidence that students with and without MHC in the same classes perceive disclosure differently and suggest that attitude and subjective norms beliefs drive these differences [21–22]. These disclosure differences also suggest the reality that stigmas exist in these introductory biology classes and may temper the climate for students with MHC.

### Do students with and without MHC make different choices about hypothetical disclosure of MHC to an instructor?

In our study, students with MHC were less likely to say they would disclose to an instructor compared to their peers without MHC. Others have documented a reluctance by students with MHC to disclose, even in the face of increasing support and awareness about MHC [13]. However, our study is the first, to our knowledge, to directly compare differences in disclosure intentions between students with and without MHC. Interestingly, we found that more than half of students with *and* without MHC indicated that their disclosure “would depend” on other factors. This indicates that students without MHC can envision barriers and benefits to disclosure of MHC, although our results suggest they may be doing so differently from students with MHC. Almost 10% of students with MHC said they would disclose. Some research has suggested that prior disclosure makes it more likely for a student to say they would disclose [24], however, negative prior experiences lead to increased reluctance to disclose in the future [28]. Given this, studying how prior disclosure impacts future disclosure intentions could help clarify the extent to which past experiences contribute to current perceptions of stigma in introductory classrooms.

### What are the differences in disclosure reasoning for students with and without MHC?

#### Attitude and subjective norms beliefs were most prevalent in shaping disclosure perceptions.

We organized our disclosure codes with the Theory of Planned Behavior (TPB) [21–23] and found that attitude and subjective norms beliefs were most salient to our participants’ intention to disclose. Attitude beliefs were often related to student perceptions about the positive outcomes of receiving an accommodation that would support their course performance. These results suggest the most prevalent benefit students were weighing was the motivation to perform well in these courses. Subjective norms beliefs were most commonly related to a perception that MHC were private; these were one of the leading rationales of students who said they would not disclose. Given that the disclosure prompt was a scenario about MHC impacting course performance, this indicates about a third of students were willing to suffer academic consequences for the sake of privacy. Our results differed slightly from the recent quantitative analysis relating TPB to instructor disclosure intentions; that study found that all three beliefs were related to intention to disclose, with subjective norms being most strongly related [24]. However, their data collection involved forced choice responses versus our open-ended prompts, making the results difficult to compare. The disclosure climate at a small private college in California may also be significantly different than at a large research-intensive institution in the southeast. We suggest that there is a need for more research on MHC disclosure guided by TPB. Specific to our study, we see a need to explore cost/ benefit tradeoffs between attitude and subjective norms beliefs such as performance and privacy. These could be explored using scenarios with various academic impacts and accommodations to better understand when students are willing to break norms for potential positive outcomes.

#### Disclosure reasoning differed by disclosure choice and MHC status.

We found few differences in disclosure reasoning among students who said yes to disclosure, regardless of MHC status. These students often indicated proactive help-seeking, talking about their intention to disclose their MHC to the instructor in advance of any problems so they could work together in the future. Very few of these students talked about concerns related to privacy. This mirrors findings by Busch and colleagues [11] that some students saw the benefits of building relationships with professors to support MHC. Although disclosure reasoning was similar among those with and without MHC, there were far fewer students with MHC who said yes to disclosure (9% of students with MHC compared to 25% without MHC), and disclosure overall was low across both groups. This aligns with reports of low rates of disclosure even as MHC support has increased [13], but also indicates that even students without MHC are reluctant to disclose. Interviewing students who are willing to disclose may provide more insight into whether they see fewer barriers, whether they see them differently, or how they cope with disclosure concerns may inform work on interventions to increase disclosure.

Students with and without MHC differed in disclosure reasoning when it came to why they said ‘no’ or ‘it would depend.’ For students with MHC, the perception that instructors may not care and the belief that the instructor would not help following disclosure was a significant hindrance to disclosure. Similarly, Busch and colleagues [11] also found that students with depression feared negative reactions from their instructors. Research has found that students who had had negative disclosure experiences in the past were less likely to view future disclosure positively [28], suggesting that past experiences could have driven perceptions in this study as well. Previous studies also found that students weigh stigma and fear of negative consequences when deciding whether to disclose MHC to instructors [11,17,18,20], and while our study found this as well, these perceptions were higher in students without MHC than those with MHC. As the incidence of MHC have risen in the student population over time, awareness has also risen about them, and there has been some reduction in perceived stigma among those with MHC [1]. Our study may have reflected these changes, with students with MHC voicing fewer subjective norms concerns compared with those without MHC; this may be because institutional accommodation processes already required them to reveal MHC, making it more of a norm for them [12]. However, the findings that those without MHC were reluctant to disclose because of subjective norms beliefs hints at the continuing negative perceptions about MHC held by peers of students with MHC. Overall, the reasons why students might be hesitant to disclose MHC hint at the ways in which students with MHC navigate in a class environment where they do not always trust instructors to help and have peers who may not understand their concerns.

### Supporting students in introductory biology with MHC

Our results highlight the high rates of MHC among undergraduate students [1,3] and how they impact perceptions of help-seeking. Although there are many institutional units to support students with MHC, instructors play an important role in creating classroom climates of support, inclusion, and belonging that can reduce perceived stigma. For example, instructors can build a positive classroom climate early in the term through inclusive class policies and syllabi that communicate empathy and support [29–30]. Instructor communication that welcomes feedback, promotes dialogue, and conveys approachability and relatability may help students feel that when they disclose a concern that the instructor will treat it with care and respect [31]. Besides supportive policies and communication, instructors may implement brief trainings in or out of class such as the 50-minute anti-stigma intervention that was found to increase student help-seeking and decrease feelings of stigma about depression by providing information, hearing from someone with depression, and being provided time to ask questions [32]. Although these instructor practices may generate positive student perceptions of classroom climate, instructors may also need professional development to combat their own biases related to MHC.

Given the high numbers of students with MHC in college classrooms, instructor training to raise self-awareness about their own potential MHC biases is needed to reduce student perceptions that instructors lack concern. Imagined intergroup contact (IIC) (i.e., structured role-play scenarios) is a simulation that can promote an individual’s positive perceptions of outgroups (such as students with MHC) and raise awareness of potential negative explicit and implicit biases toward these groups [33]. IIC has also been found to reduce the anxiety surrounding potential interactions with outgroups, which may promote more positive communication strategies and decrease avoidance behaviors [34]. Integrating IIC into instructor training could help instructors navigate conversations about MHC accommodations in ways that convey more support and respect, and with a willingness to provide assistance. This focus on how instructors can support students with MHC must be coupled with institutional support for faculty who may also be experiencing their own MHC and burnout.

### Limitations and future work

The sample for this study was drawn from a single institution, constraining our ability to generalize to the larger population of students at other institutions. This study was also conducted only in introductory biology courses, which are a unique sub-population at our institution and further bound any inferences we could make. Studies in other classes and at other institutions, particularly with a more representative sample of students from historically marginalized groups, would provide additional evidence about whether the differential student reasoning for disclosure are generalizable to a broader population. This study also did not disambiguate the data by instructor or class section, which may be a focus of future work. It is likely that there were different rates of student disclosure among the courses and classroom observations could attempt to identify features of classrooms that could be emulated in other courses.

There was a potential for authoritative bias in the results due to the different positionalities of the coders in this study. Steps were taken to provide adequate training for M.S. in qualitative methods and discussions were held prior to coding about the need to have equal voice in coding decisions. In discussions about coding disagreements, strength of argument was prioritized, and final code decisions were not made more often by the more senior coder, suggesting a relatively equal contribution to the coding results.

This study focused on students’ intent to disclose versus actual disclosure. Although theory suggests a tight relationship between the two, actual disclosure was not studied, nor could it be given the lack of MHC in half the respondent population. Asking students without MHC to imagine a scenario where they have MHC may not represent how they would actually act. However, our results do hint at the differential assumptions held by each group, which highlight a need to better understand not just reasoning behind disclosure, but also where those ideas originate. A study on this could probe the relative impacts of prior disclosure, perceptions of stigma, motivation to perform well in a course, and instructor trust [35], among others.

### Conclusion

Given the high numbers of students with MHC at undergraduate institutions [1] and the negative impacts MHC can have on their collegiate performance, it is important to understand how their unique perspectives and experiences may differentially impact their success. This study suggests that students with MHC have different perceptions compared to their peers without MHC about disclosing an MHC to an instructor. Students with MHC indicated negative perceptions of instructor reactions to disclosure that could act as barriers to help-seeking. Their peers without MHC were concerned more about privacy and performance when considering the same hypothetical disclosure question. This suggests students with MHC navigate in an environment where they may assume less understanding from their instructors and interact with peers who think MHC should be private. Future research should focus on factors to bridge these classroom perceptual differences to ensure an environment where students with MHC may disclose their needs without fear of negative judgment or inadequate assistance.

## Supporting information

S1 Table Survey questions and associated purpose for the study.
(DOCX)

S2 Appendix Data underlying the results.
(XLS)

S3 Appendix Additional exemplar quotes for codes.
(XLSX)
